# Critical analysis of datasets for sign language translation

**DOI:** 10.3389/frai.2026.1743223

**Published:** 2026-05-29

**Authors:** Bittor Alkain, Adrián Núñez-Marcos, Carlos Escolano, Laura Docío-Fernández, Olatz Perez-de-Viñaspre, Gorka Labaka

**Affiliations:** 1 HiTZ Center - Ixa, University of the Basque Country UPV/EHU, San Sebastián, Spain; 2 Barcelona Supercomputing Center, Barcelona, Spain; 3 atlanTTic Research Center, University of Vigo, Vigo, Spain

**Keywords:** dataset analysis, datasets, Machine Translation (MT), natural language process (NLP), overfitting, Sign Language Translation, sign languages, visual-language models

## Abstract

**Introduction:**

In recent years, significant progress has been made in Machine Translation (MT), including multilingual and low-resource settings. However, Sign Language Translation (SLT) remains underdeveloped, largely due to the scarcity of high-quality datasets and the overreliance on a few small, widely used benchmarks. This study aims to critically assess the datasets most commonly used in SLT research to determine whether their characteristics may lead to overfitting and misleading evaluation results.

**Methods:**

We then conduct a detailed empirical study comparing training and test set similarity for PHOENIX14T, CSL-Daily, and LSE-Health. Using both gloss-based (TwoStream-SLT) and gloss-free (GFSLT-VLP) models, we evaluate the extent to which models memorize training data and how this affects BLEU scores.

**Results:**

Our analysis reveals that PHOENIX14T exhibits substantial overlap between training and test sets, leading to inflated BLEU scores and can even mask signs of overfitting. CSL-Daily shows less overlap and more robust generalization. We also show that a small subset of “training-like” sentences disproportionately contributes to BLEU scores.

**Discussion:**

We recommend that future SLT research move away from overused benchmarks and adopt larger, more diverse datasets such as How2Sign, CSL-News, and FLEURS-ASL. We also advocate for a shift toward gloss-free approaches and more careful interpretation of evaluation metrics, especially in low-resource settings.

## 1 Introduction

During the past decades, the growing research in the field of Machine Translation (MT) has led to an increasing interest in the automatic translation of sign languages (SLs). In contrast to the MT task, which is a text-to-text mapping, Sign Language Translation (SLT) aims at transforming a sign language utterance (stored as a video) into a spoken language text with the same content and meaning. The reciprocal translation from a spoken language text to a sign language utterance is called Sign Language Production (SLP) and requires the synthesis of an avatar or a photo-realistic signer. SLT has received much more attention than SLP in the last decade.

While the MT task has seen huge improvements, leading to the development of well-known models (Liu et al., 2020; Costa-jussà et al., 2022), advancements in SLT have been delayed due to various reasons: (i) the increased difficulty of working with videos (more storage and computing resources required, and the complexity of extracting suitable features), (ii) the limited number and size of datasets; the general lack of aligned annotations and, when present, their lack of standardized notation.

In contrast to MT datasets, annotating an SLT dataset requires proficient SL users, and the annotation process takes much more time than its spoken language counterpart due to the visual nature of SL discourse. This is even more demanding if glosses^1^ are also annotated, as no globally recognized criteria exist to define them. Hence, platforms such as Amazon Mechanical Turk are not suitable to address this issue.

Moreover, there is a privacy concern for SL users being recorded, as their faces can be recognized. Although works such as Rust et al. (2024) attempt to address this issue by blurring the signer's face, they conclude that the face is an important feature for SLT. This makes data collection or the creation of new datasets more difficult, as consent forms are required at every step.

In this work we compiled the characteristics of the available datasets for SLT and analyze the two of them that are more frequently used in the recent literature, namely, PHOENIX14T (Camgoz et al., 2018) and CSL-Daily (Zhou et al., 2021). We also compare them with the recently published LSE-Health dataset (Alba-Castro et al., 2023). Hence, we make the following key contributions to advance the field of Sign Language Translation (SLT):

- Comprehensive dataset landscape: to the best of our knowledge, we provide the most extensive and up-to-date compilation of publicly available SLT datasets, detailing their linguistic coverage, annotation schemes, and accessibility. This resource aims to serve as a reference point for future research and reproducibility.
- Critical benchmark analysis: we conduct the first in-depth examination of the two most widely used SLT benchmarks—PHOENIX14T and CSL-Daily—revealing structural biases and overlap patterns that can lead to overfitting and inflated BLEU scores. We also include a comparative analysis with the recently introduced LSE-Health, which broadens the linguistic scope of the study by incorporating Spanish Sign Language. Despite being similar in size to PHOENIX14T, LSE-Health provides broader domain, which enables a more informative contrast with the most widely used benchmark.
- Quantitative evidence of overfitting: through a novel “training-likeness" metric and cumulative BLEU analysis, we demonstrate how a small subset of highly similar sentences disproportionately drives evaluation results, challenging the reliability of current benchmarking practices.

## 2 Datasets

In this section, we present a curated overview of publicly available datasets for Sign Language Translation (SLT). To identify relevant resources, we conducted a systematic search using Google Scholar with the query “sign language dataset." To broaden the search, we examined survey papers, as well as the content and citations of the papers initially identified, and we also reviewed papers with publicly available code listed on the Papers With Code platform (https://paperswithcode.com/). We included only those datasets that are publicly accessible, contain sentence-level aligned sign language video and spoken language text (rather than isolated sign–word pairs), and offer approximately 10 or more hours of sign language video content.

Table 1 summarizes the datasets that meet these criteria. Each dataset is characterized by a set of attributes that facilitate comparison and highlight their suitability for SLT research. The table is organized first by sign language (SignL), and then by the corresponding spoken language (SpL), reflecting the most common structure of SLT datasets.

**Table 1:** Publicly available SLT datasets satisfying our inclusion criteria, grouped by sign language (SignL) and spoken language (SpL), with key characteristics relevant to SLT research.

Dataset	SignL	SpL	Hours	Signs	Chroma	One & Front	Segm.	Vocab.	Signers	Source	Glosses	Cites
YouTube-ASL Uthus et al. (2023)	ASL	EN	984	–	Mixed	Mixed	610K	60K	2.5K	Web	✗	73
OpenASL Shi et al. (2022)	ASL	EN	288	–	Mixed	Mixed	98K	33K	220	Web	Partial	85
How2Sign Duarte et al. (2021)	ASL	EN	80	–	✓	✓	35K	16K	11	Lab	Partial	336
DailyMoth-70h Rust et al. (2024)	ASL	EN	70	–	✓	✓	48K	19K	1	Web	✗	31
Auslan-Daily Shen et al. (2023)	ASF	EN	45	–	✗	✗	25K	14K	67	TV & Web	✗	29
BOBSL Albanie et al. (2021)	BSL	EN	1,467	>2,281	✗	✓	1.2M	78K	37	TV	✗	109
BSL corpus Schembri et al. (2013)	BSL	EN	125	7,332	✓	✗	–	–	249	Lab	✓	196
ISLTranslate Joshi et al. (2023)	ISL	EN	55	–	✓	✓	31K	11K	–	Web	✗	16
iSign Joshi et al. (2024)	ISL	EN	252	–	✓	✓	118K	40K	–	TV & Web	✗	10
Phoenix-2014T Camgoz et al. (2018)	DGS	DE	11	1,066	✓	✓	8.2K	3K	9	TV	✓	967
SIGNUM (von Agris and Kraiss 2010)	DGS	DE	55	450	✓	✓	780	1K	25	Lab	✓	43
Public DGS corpus Hanke et al. (2020)	DGS	DE/EN	50	–	✓	✓	70K	23K	327	Lab	✓	80
SWISSTXT-NEWS Camgoz et al. (2021)	DSGS	DE	9	–	✗	✗	6K	10K	–	TV	✗	55
SRF23 Müller et al. (2023)	DSGS	DE	437	–	✓	✓	233K	–	4	TV/Lab	✗	27
LSE-Health Alba-Castro et al. (2023)	LSE	ES	11	~2,000	✓	✓	7.7K	6K	10	Lab	Partial	0
LSA-T Dal Bianco et al. (2022)	LSA	ES	21	–	✗	✗	14K	7K	103	Web	✗	14
Corpus NGT Crasborn and Zwitserlood (2008)	NGT	NL	17+	–	✓	✓	–	–	24+	Lab	✓	169
VRT-NEWS Camgoz et al. (2021)	VGT	NL	9	–	✗	✗	7K	6K	–	TV	✗	55
Mediapi-RGB Ouakrim et al. (2024)	FSL	FR	86	–	✗	✗	50K	35K	>10	TV	✗	6
Corpus FINSL Salonen et al. (2020)	FinSL	FI	14	814	✓	✗	4K	7K	21	Lab	✓	17
Elementary23 Voskou et al. (2023)	GSL	EL	71	–	✓	✓	29K	23K	9	Lab	✗	14
Elementary23-SLT Voskou et al. (2023)	GSL	EL	–	–	✓	✓	8K	8K	9	Lab	✗	14
GSL Adaloglou et al. (2021)	GSL	EL	9	310	✓	✓	310 (10K)	–	7	Lab	✓	305
AzSLD Alishzade and Hasanov (2024)	AZSL	AZ	65	–	✓	✓	500	–	43	Lab	✓	2
CSL-Daily Zhou et al. (2021)	CSL	ZH	23	2,000	✓	✓	8K (20K)	2K	10	Lab	✓	317
CE-CSL Zhu et al. (2024)	CSL	ZH	10	–	✗	✓	6K	3K	12	Lives	✓	4
SCOPE Liu Y. et al. (2024)	CSL	ZH	72	–	✓	✓	59K	5K	12	Lab	✓	6
CSL-News Li et al. (2025)	CSL	ZH	1,985	–	✓	✓	751K	5K	–	TV	✗	20
TVB-HKSL-News Niu et al. (2024)	HKSL	ZH	16	6K	✓	✓	7K	6K	2	TV	✓	7
KETI Ko et al. (2019)	KSL	KO	28	524	✓	✓	14.6K	3K	14	Lab	✓	299
SSL Kim et al. (2024)	KSL	KO	–	–	Mixed	✓	–	–	–	Phone	✓	1
E-TSL Öztürk and Keles (2024)	TSM	TR	24	–	–	✓	–	7K	11	Lab	✗	2
YouTube-SL-25 Tanzer and Zhang (2024)	>25	?	3,207	–	Mixed	Mixed	–	–	3K	Web	✗	14
SP-10 Yin et al. (2021)	10	10	14	–	✓	✓	11K	16K	79	Lab	✓	139
AfriSign Gueuwou et al. (2023)	6	EN	152	–	✓	✓	29K	20K	–	Web	✗	16
SwissSLi Jiang et al. (2024)	3	3	30	–	✓	✓	16K	–	24	TV	✗	2

The number of cites were extracted from Google Scholar on October 22, 2025. The content of each of the columns is explained in Section 2.

The total duration of sign language video content is reported in hours, while the number of unique signs—when available—provides insight into the dataset's lexical richness. We also indicate whether the background is chromatic and uncluttered, a factor that can affect visual feature extraction. In cases where the dataset includes both controlled and uncontrolled settings, we label it as “Mixed."

Another important aspect is whether the dataset features a single signer positioned frontally (*One & Front*), which can simplify model training. The number of sentence pairs (Segm.) and the vocabulary size of the spoken language side (Vocab.) are also included to assess the dataset's scale and linguistic diversity.

We report the number of signers involved in the recordings, as well as the source of the data—whether it was collected in a lab, scraped from the web, recorded from TV broadcasts, or captured via mobile devices. The presence of gloss annotations is noted, with “Partial" indicating incomplete coverage. We also specify the number of citations it has received (as of October 22, 2025), and the year of publication.

To support reproducibility and further exploration, we compiled direct download links for all datasets in a public GitHub repository: https://github.com/olatz87/SLT-Datasets/blob/main/README.md.

The datasets compiled in Table 1 reveal a striking diversity in terms of size, structure, and annotation quality. While a few datasets, such as YouTube-SL-25 and BOBSL, offer thousands of hours of video and hundreds of thousands of sentence pairs, many others remain relatively small, often under 50 h of content. Only a subset of datasets include gloss annotations, and even fewer provide them comprehensively. This scarcity of glosses is particularly evident in large-scale datasets, where the cost and complexity of annotation make such efforts impractical. Additionally, the datasets vary widely in terms of signer diversity, recording conditions, and domain coverage, which has important implications for model generalization and evaluation. These disparities underscore the need for careful dataset selection and highlight the challenges of building robust SLT systems.

## 3 A critical look at SLT benchmarks

While the previous section highlighted the growing availability and diversity of SLT datasets, a closer look at recent literature reveals a strong reliance on a small subset of these resources—most notably, PHOENIX14T (Camgoz et al., 2018) and CSL-Daily (Zhou et al., 2021). These datasets have become *de facto* benchmarks in the field, frequently used to train and evaluate new models (Fu et al., 2025; Gong et al., 2024; Kim et al., 2025; Chen et al., 2024; Wong et al., 2024; Ye et al., 2024; Moon et al., 2025; Liu Z. et al., 2024). However, this widespread adoption raises important concerns about overfitting and the generalizability of results.

In particular, these benchmark datasets are relatively limited in size, especially when compared to the complexity of the SLT task. For instance, PHOENIX14T contains just over 8,000 sentence pairs, yet it is often used as the sole training source in many studies. While this may be justified by the linguistic uniqueness of each sign language—making cross-dataset training non-trivial—it also raises questions about how much meaningful knowledge models can extract from such constrained data. The consistently high BLEU scores reported on these datasets suggest that models may be learning dataset-specific patterns rather than generalizable translation capabilities. This phenomenon is reminiscent of the overfitting issues observed with the MNIST dataset in the computer vision community.

In this section, we investigate this hypothesis by analyzing the extent to which overfitting may be influencing reported performance. We begin by describing the datasets and the metric proposed for our analysis, and examining the latter's value for each dataset (Section 3.1), then we present the two models considered in this work and their behavior on the presented datasets (Section 3.2), and finally assess the impact of “training-like" sentences on evaluation metrics (Section 3.3).

### 3.1 Dataset intrinsic overlap

In datasets with limited domain coverage, it is not uncommon for the training and test splits—despite being signer-independent—to contain overlapping content. This overlap may include repeated phrases or even entire sentences, particularly in structured domains such as weather forecasts. As a result, evaluation scores can be artificially inflated, since models may benefit from having already seen highly similar examples during training. This effect can obscure the true generalization capabilities of a model and, in extreme cases, may turn the task into a form of pattern matching rather than genuine translation.

To disentangle these dataset-intrinsic effects from model behavior, we first analyze the degree of overlap present in commonly used SLT benchmarks. In particular, we focus on three datasets that vary in size, domain, and annotation quality: PHOENIX14T, CSL-Daily, and LSE-Health. These datasets represent different usage patterns in the literature and allow us to contrast high-resource and low-resource scenarios.

PHOENIX14T (short for RWTH-PHOENIX-Weather 2014T) consists of 8,257 sentence pairs derived from German TV weather forecasts. Each pair includes a video in German Sign Language (DGS) and its corresponding spoken German sentence. The dataset is split by forecast segments, which results in many sentences appearing in both the training and test sets, even if their video counterparts are not identical—especially in the case of formulaic expressions such as greetings and closings.^2^

CSL-Daily is a Chinese Sign Language dataset comprising 20,653 sentences across various everyday scenarios. Its structure is similar to PHOENIX14T, but it offers a larger training set (18,400 sentences) and a broader range of topics. This increased scale and diversity are expected to reduce the likelihood of repeated content across splits, providing a useful contrast to more constrained datasets.

To complement these widely used benchmarks, we included LSE-Health, a recently released dataset focused on health-related content in Spanish Sign Language (LSE). It contains 7,738 sentence pairs recorded by a mix of deaf signers and interpreters for the Galician Health Department's YouTube channel. Although it is similar in size to PHOENIX14T, LSE-Health has a richer vocabulary and a more diverse range of signers. It is partially annotated with glosses and incorporates both topic- and signer-dependent splits.

Together, these datasets provide a balanced testbed for analyzing intrinsic overlap under different conditions. PHOENIX14T and CSL-Daily account for the majority of existing SLT literature and therefore serve as representative benchmarks, while LSE-Health broadens the linguistic and domain coverage of the analysis. At the same time, they cover spoken languages from distinct language families—German (Germanic), Chinese (Sino-Tibetan) and Spanish (Romance)—and sign languages with different linguistic and cultural backgrounds.

To quantify the degree of intrinsic overlap in each dataset, we perform a similarity analysis between the training set and the reference translations in the test set. This allows us to establish a dataset-level baseline of how “training-like" the evaluation data is, independently of any model behavior.

We define the *training-likeness* score of a sentence as its maximum string similarity to any sentence in the training set. Formally, for a sentence *s*, we compute:


$$\text{training-likeness}\left(s\right)=\underset{t\in T}{\max}\text{sim}\left(s,t\right)$$

where *T* is the set of training sentences and sim is a string similarity function. In practice, we use the fuzz.ratio function from the RapidFuzz library.^3^ A score of 100 indicates an exact match with a training sentence, while lower values reflect decreasing lexical similarity.

To illustrate this measure, Table 2 presents several sentences from the PHOENIX14T test set, along with their closest counterpart in the training set and the corresponding similarity score, that is, the *training-likeness* of the test set sentences. These examples are intended to provide an intuitive reference for how this metric behaves across different levels of similarity, ranging from loosely related sentences to near-exact matches.

**Table 2:** Example sentences from the PHOENIX14T test set, together with their most similar training-set sentence and the similarity score between them (marked in bold), that is, the training-likeness of the test set sentences (metric explained in Section 3.1).

Example element	German sentence	English translation
Example A		
Reference	*und es wird auch wieder schneien oberhalb von dreihundert metern meist so am nachmittag kräftig vom saarland bis nach berlin hoch*	“And it will snow again above 300 m mostly in the afternoon heavily from Saarland up to Berlin"
	↑↓ Similarity score: **58.1**	
Most similar training-set sentence	*dazu kann es aber glatt werden oberhalb von dreihundert metern rechnen wir in den mittelgebirgen heute nacht mit schnee*	“However, it can be slippery above 300 m we expect snow in the low mountain ranges tonight"
Example B		
Reference	*dabei breiten sich heute nacht von nordwesten regenfälle bis in die mitte aus*	“Rainfall will spread from the northwest to the center tonight"
	↑↓ Similarity score: **66.7**	
Most similar training-set sentence	*die gewitter breiten sich heute nacht von südwesten aus*	“The thunderstorms will spread from the southwest tonight"
Example C		
Reference	*am freitag ist es im südwesten zunächst freundlich*	“On Friday it will initially be pleasant in the southwest"
	↑↓ Similarity score: **74.3**	
Most similar training-set sentence	*am tag ist es im westen und nordwesten meist freundlich*	“During the day it will be mostly pleasant in the west and northwest"
Example D		
Reference	*der wind weht schwach bis mäßig am meer auch frisch*	“The wind blows light to moderate, also fresh at the sea"
	↑↓ Similarity score: **91.4**	
Most similar training-set sentence	*der wind weht schwach bis mäßig an der see auch frisch*	“The wind blows light to moderate, also fresh at the sea"

Each sentence is accompanied by an English translation. PHOENIX14T: Camgoz et al., 2018; CSL-Daily: Zhou et al., 2021; LSE-Health: Alba-Castro et al., 2023

**Figure 1:**
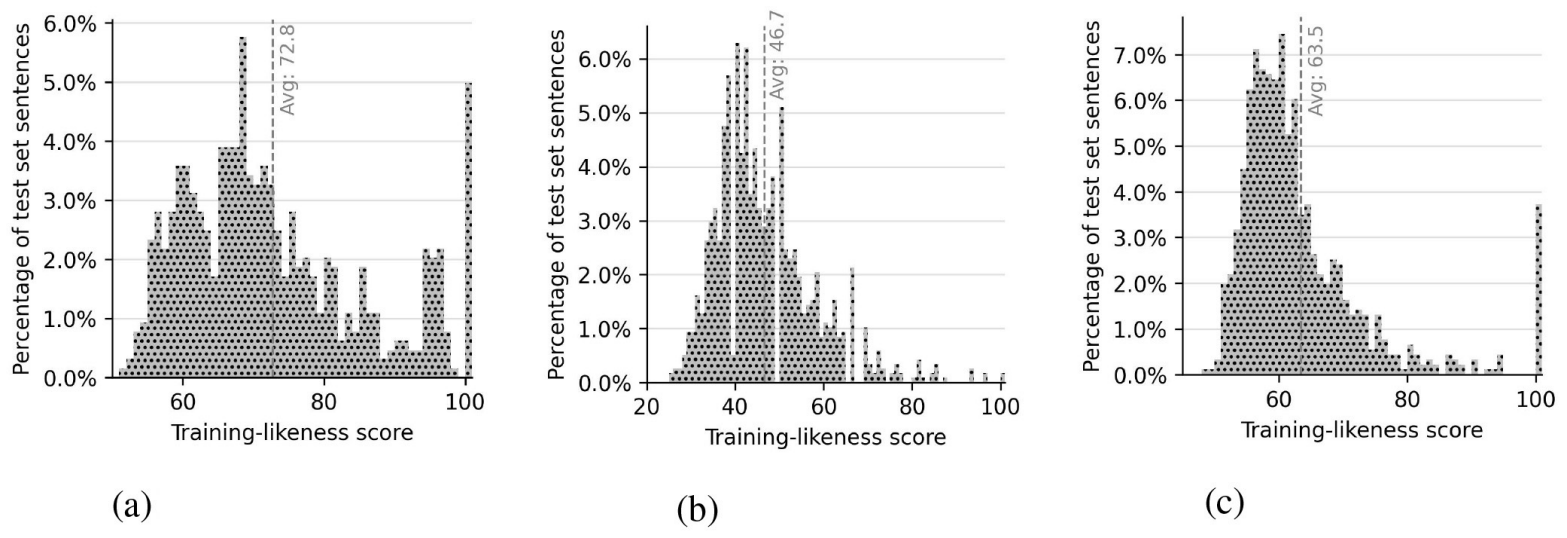
**(a)** PHOENIX14T. **(b)** CSL-Daily. **(c)** LSE-Health. Histograms showing the *training-likeness* (metric explained in Section 3.1) of reference translations for test sets from different datasets. The vertical line in each plot marks the average *training-likeness* over the full test set. Spikes at the maximum score (100) correspond to exact matches with training sentences.

Histograms in Figure 1 show the *training-likeness* of the test set reference translations for all three datasets. While score distributions are not directly comparable across datasets—since they depend on factors such as the target language—they still provide insight into each dataset's design. In particular, the last bar corresponds to exact matches (score 100), where PHOENIX14T clearly stands out: around 5% of its test sentences fall into this category, with many others scoring close to 100. Importantly, this should not be interpreted as strict data contamination, since the corresponding input videos are not identical across splits; rather, it reflects the presence of repeated or highly formulaic translations. This is consistent with the dataset characteristics explained at the beginning of the current section.

Based on the average score over the full test set, PHOENIX14T exhibits the highest degree of overlap (78.2), followed by LSE-Health (63.5) and CSL-Daily (46.7), already suggesting substantial differences in the intrinsic similarity between training and test splits. Beyond these averages, the shape of the distributions provides additional insight. PHOENIX14T displays a noticeably flatter histogram, with a lower peak and a heavier tail toward high similarity values. This indicates that a relatively large proportion of test sentences have medium-to-high similarity with training examples, rather than being concentrated around a narrow range. In contrast, LSE-Health shows a more concentrated distribution, with fewer high-similarity cases, while CSL-Daily exhibits the most peaked distribution and the lowest presence of high-similarity examples.

Taken together, these patterns suggest that PHOENIX14T contains a broader spread of training-like sentences, including a significant number of highly similar or near-duplicate examples, whereas such overlap is progressively less pronounced in LSE-Health and especially in CSL-Daily. This reinforces the idea that the degree of dataset-intrinsic overlap varies substantially across benchmarks and may play a key role in shaping evaluation outcomes.

### 3.2 Model behavior: memorization vs. generalization

Having characterized the degree of intrinsic overlap in each dataset, we now turn to analyzing how current SLT models behave under these conditions. In particular, we examine whether model outputs reflect the underlying training-test similarity or whether they exhibit additional tendencies toward memorization.

To this end, we consider two representative SLT architectures. The first is GFSLT-VLP (Zhou et al., 2023), a gloss-free architecture based on visual-language pretraining. It combines a visual encoder and a text decoder trained using contrastive language-image learning (CLIP, Radford et al., 2021) and masked self-supervised objectives. GFSLT-VLP was the leading gloss-free model prior to the release of Sign2GPT (Wong et al., 2024), and is particularly relevant for datasets without gloss annotations.

For PHOENIX14T, we trained GFSLT-VLP using the authors' publicly available code, achieving BLEU scores closely aligned with those reported in the original paper (BLEU: 20.27 vs. 21.44). For CSL-Daily, we used the authors' pre-trained models, which also reproduce the published performance (BLEU: 10.98 vs. 11.00). Due to the limited gloss coverage in LSE-Health, this dataset is evaluated exclusively with the gloss-free GFSLT-VLP model.

As a complementary approach, we also evaluate TwoStream-SLT (Chen et al., 2022), a gloss-based architecture that processes both raw RGB video and keypoint sequences extracted by a pose estimation model. This type of architecture is widely used in gloss-annotated settings and remains state-of-the-art in that category.

For our experiments, we rely on the pre-trained models provided by the authors in their official GitHub repository.^4^ These correspond to the configurations used in the original publication, and we do not retrain them.

It is worth noting that the version of the CSL-Daily dataset used in our experiments—provided directly by USTC—differs slightly from the one used in the original paper. However, these differences are minor and are not expected to significantly affect the reported results.

The *training-likeness* metric introduced in Section 3.1 enables a direct comparison between the similarity distributions of reference translations and model-generated outputs. Intuitively, if a model tends to reproduce sentences seen during training, its outputs will exhibit higher average *training-likeness* than the references themselves.

Although this metric does not account for semantic variation such as paraphrasing or synonymy, this limitation is consistent with our objective: we deliberately focus on surface-level lexical overlap as an indicator of memorization of training data by current SLT models.

**Figure 2:**
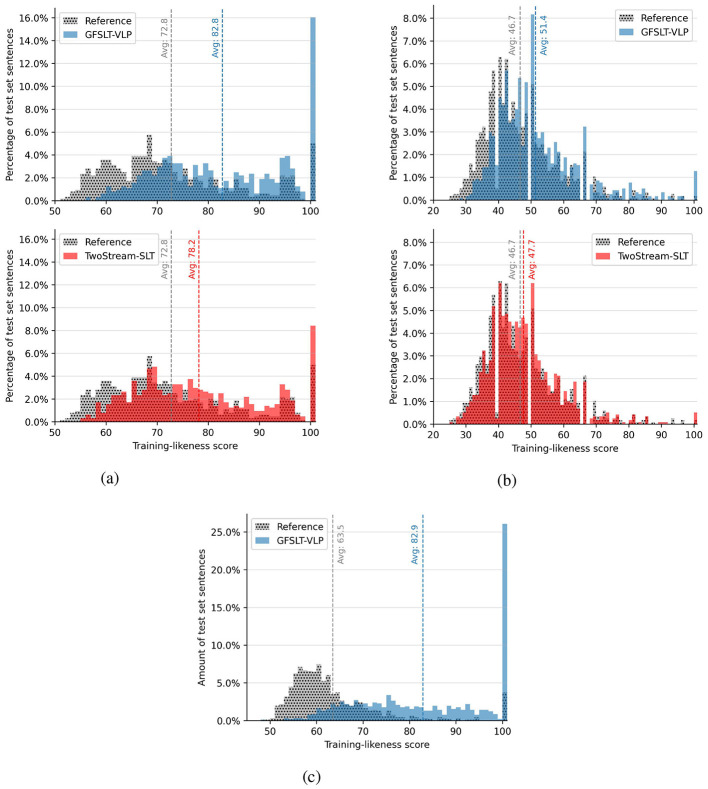
**(a)** PHOENIX14T. **(b)** CSL-Daily. **(c)** LSE-Health. Histograms showing the *training-likeness* (metric explained in Section 3.1) of generated translations (colored) for test sets from different datasets, compared to the same metric for reference translations (gray and dotted). The two vertical lines in each plot mark the average *training-likeness* over the full test set for system outputs and references. When the average for automatic translations is notably higher than for references, this indicates a tendency toward overfitting. Spikes at the maximum score (100) correspond to exact matches with training sentences. Only one histogram is shown for LSE-Health, since it can only be used with gloss-free models.

Figure 2a illustrates this effect for the PHOENIX14T dataset. The GFSLT-VLP model shows a pronounced bias toward generating “training-like” sentences: 16% of its outputs are exact matches to training examples. In contrast, only 5% of the reference translations appear verbatim in the training set. This suggests that the model is not generalizing well and may be relying heavily on memorization. The TwoStream-SLT model, which incorporates gloss supervision, exhibits a lower degree of this behavior, consistent with its stronger performance and more structured input representation.

This memorization behavior may reflect a reduced reliance on the visual input during decoding. This possibility is consistent with prior findings from interpretability analyses on a different sign language dataset, which show that, after initially attending to the video, models can become dominated by self-attention over previously generated tokens, in some cases enabling sentence prediction with minimal visual grounding (Dal Bianco et al., 2024).

The CSL-Daily dataset presents a contrasting picture (Figure 2b). Here, both the reference and generated sentences exhibit low similarity to the training set, and the models rarely produce exact matches. This is likely due to the dataset's larger size (18,400 training sentences vs. 7,096 in PHOENIX14T) and broader topical coverage, which reduce redundancy and encourage generalization.

In the case of LSE-Health, a low-resource dataset, the GFSLT-VLP model exhibits extreme overfitting. As shown in Figure 2c, over 25% of its outputs are exact copies of training sentences. This aligns with the dataset's small size and wider domain. The model appears unable to learn meaningful translation patterns and instead defaults to memorization. This is further corroborated by the low BLEU score (4.3) and manual inspection of the outputs, which reveal a lack of coherent or contextually appropriate translations.

**Figure 3:**
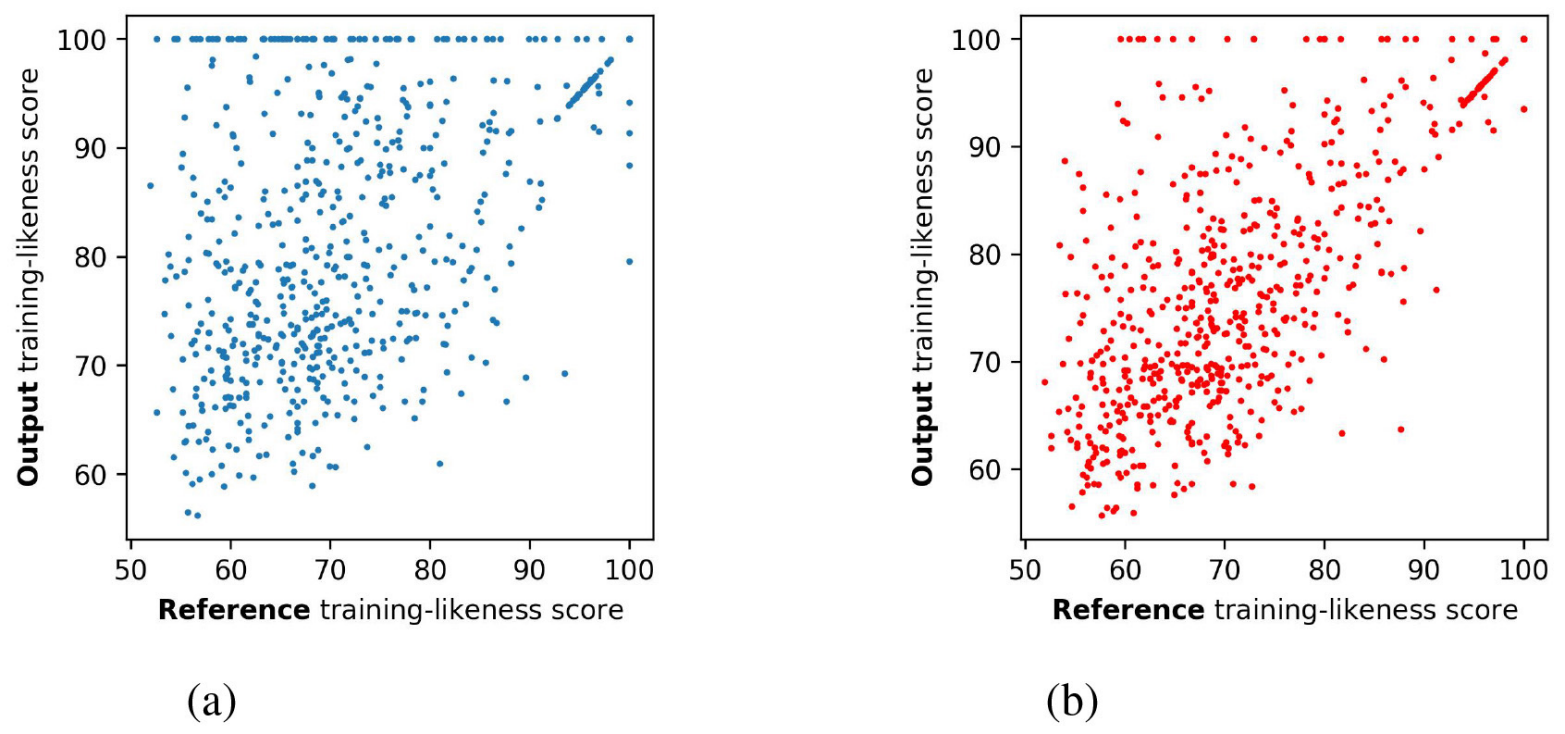
**(a)** GFSLT-VLP. **(b)** TwoStream-SLT. Individual *training-likeness* (metric explained in Section 3.1) of generated translations for the PHOENIX14T test set compared to the same metric for reference translations. Points above the diagonal correspond to outputs that are more similar to the training set than their references, revealing a tendency toward memorization.

To get a closer look at the comparison between the reference translations and the outputs, Figure 3 shows the individual *training-likeness* for each sentence in the test set, only for PHOENIX14T. As expected, since outputs get higher scores than references, the top-left triangles of the plots are more populated. The visible line of dots at the 100-point mark for the GFSLT-VLP outputs (Figure 3a) shows that the model tends to reproduce a training example even for sentences whose reference translation is not similar to any training example. As a result of the better performance of the gloss-based model (Figure 3b), it gets a bigger dot concentration around the diagonal of equal *training-likeness* of output and reference.

To contextualize these findings, we considered a hypothetical baseline for text-to-text translation: a system that retrieves the most similar sentence from the training set and outputs its translation. Such a system could achieve deceptively high BLEU scores if the test set closely resembles the training set. However, replicating this baseline for video-to-text translation is non-trivial, as it requires a method to compare videos based on their semantic content. Our attempts to implement such a system were unsuccessful.

These results underscore the importance of analyzing dataset characteristics when interpreting evaluation metrics. A high BLEU score may not reflect true translation quality if it is driven by memorization rather than generalization. In the next section, we quantify the contribution of these “training-like” sentences to the overall BLEU score.

### 3.3 Dissecting BLEU: the impact of training-like sentences

Having established that a significant portion of test set sentences in PHOENIX14T are highly similar or even identical to those in the training set, we now turn to quantifying the influence of these “training-like” sentences on the overall BLEU score. Specifically, we aim to determine how much of the reported performance can be attributed to these easier, potentially memorized examples.

To this end, we first sort the reference sentences in the test set by their *training-likeness* scores, as defined in Section 3.1, from least to most similar. We then compute the BLEU score for the model-generated outputs over progressively larger subsets of the test set, starting with the least similar sentences and incrementally including more “training-like” ones. This cumulative evaluation allows us to observe how BLEU evolves as increasingly familiar content is introduced.

**Figure 4:**
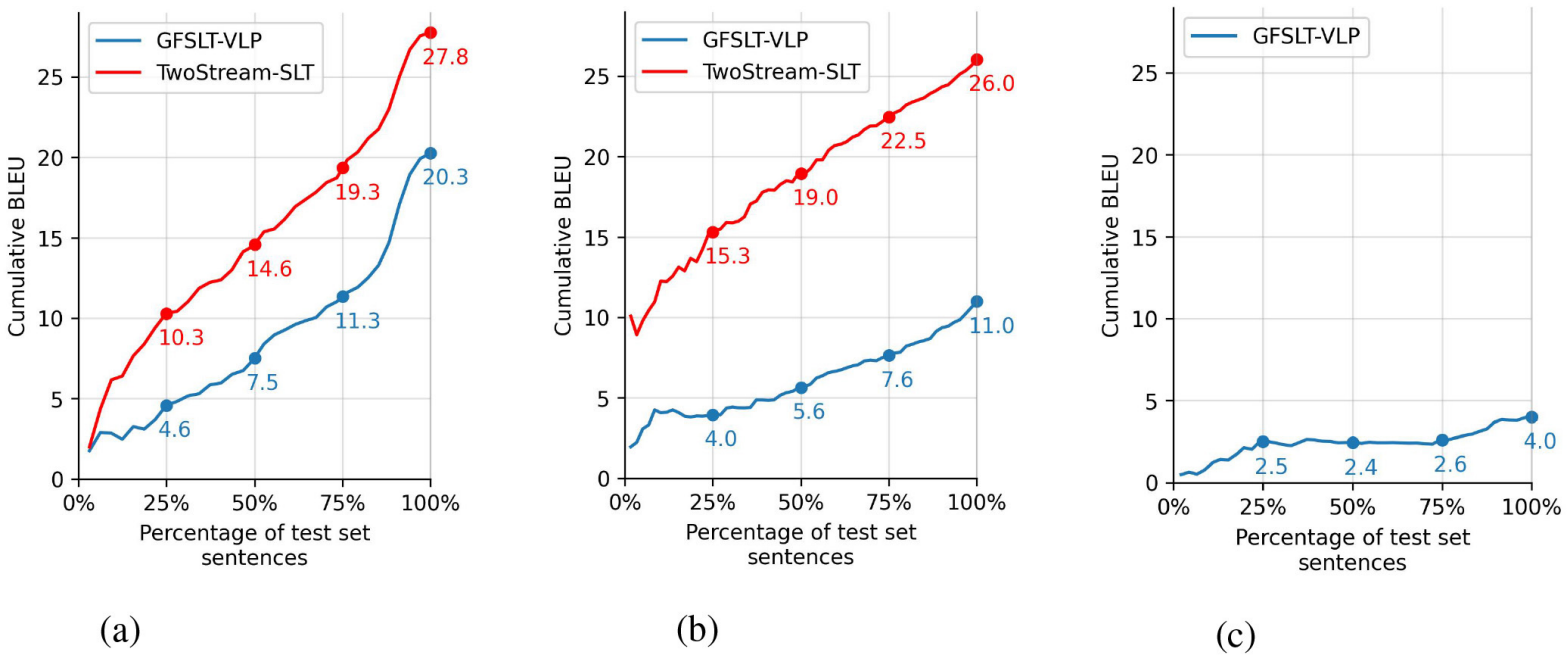
**(a)** PHOENIX14T. **(b)** CSL-Daily. **(c)** LSE-Health. Cumulative BLEU score for an increasing number of sentences from the test set, sorted by increasing *training-likeness* (metric explained in Section 3.1), for different datasets. Sharp BLEU increases toward the end of the curves indicate that highly training-like sentences disproportionately contribute to the final score. Only one line is shown for LSE-Health, since it can only be used with gloss-free models.

Figure 4 presents the results of this analysis. For PHOENIX14T (Figure 4a), we observe a strong correlation between *training-likeness* and BLEU score. The inclusion of the top 15%–20% most “training-like” sentences significantly boosts the final score. In the case of GFSLT-VLP, the BLEU score nearly doubles when the most similar 30% of sentences are included. This finding underscores the extent to which high BLEU scores on this dataset may be driven by memorization rather than true generalization.

For CSL-Daily (Figure 4b), the effect is less pronounced. While there is still a positive correlation between *training-likeness* and BLEU, the increase is more gradual, and the final score is less dependent on a small subset of highly similar sentences. This aligns with our earlier observation that CSL-Daily contains fewer repeated phrases and exhibits greater linguistic diversity.

In contrast, the LSE-Health dataset (Figure 4c) presents a different challenge. As discussed in Section 3.2, the GFSLT-VLP model fails to generalize and instead resorts to copying almost random training sentences. As a result, the overall translation quality is so poor that BLEU becomes largely insensitive to meaningful differences. Nevertheless, the same pattern observed in other datasets persists, as the last 25% of sentences raises the final BLEU score. However, this relative gain occurs at such a low absolute level that it does not represent a practically meaningful improvement in translation quality.

In addition to the cumulative BLEU, Table 3 reports the independent BLEU score for each test set quartile, with quartiles defined by *training-likeness* as before. The scores mirror the patterns shown in the cumulative BLEU analysis. Most significantly, for PHOENIX14T, the scores for both models differ by roughly 52 points between Q1 (the least “training-like” quartile) and Q4. In fact, Q4 is the quartile with the shortest average sentence length in PHOENIX14T, which gives it a lower weight in the overall BLEU score. However, because of the massive difference in scores, it has a disproportionately large influence on the final score.

**Table 3:** BLEU scores for test-set sentences split into quartiles by increasing *training-likeness* (metric explained in Section 3.1).

Model	PHOENIX14T				CSL-Daily				LSE-Health			
	Q1	Q2	Q3	Q4	Q1	Q2	Q3	Q4	Q1	Q2	Q3	Q4
GFSLT-VLP	4.6	12.2	19.1	56.0	4.0	7.4	11.8	22.2	2.5	2.3	2.8	8.3
TwoStream-SLT	10.3	20.5	29.5	62.5	15.3	23.0	31.0	38.9	–	–	–	–

Only the numbers for GFSLT-VLP are shown for LSE-Health, since it can only be used with gloss-free models.

These results highlight the importance of interpreting BLEU scores in the context of dataset characteristics. In particular, they caution against over-reliance on benchmark datasets like PHOENIX14T, where high performance may be partially explained by the presence of repeated content. Future evaluations should consider complementary metrics and more diverse datasets to better assess model generalization.

## 4 Conclusions

In this work, we have presented a comprehensive analysis of publicly available datasets for Sign Language Translation (SLT), with a particular focus on their suitability for training and evaluating modern SLT models. In Section 2, we surveyed the landscape of SLT datasets, highlighting their diversity in terms of size, annotation quality, and linguistic coverage. In Section 3, we critically examined the two most widely used benchmarks—PHOENIX14T and CSL-Daily—revealing potential overfitting issues that may compromise the generalizability of reported results.

Our findings suggest that the high BLEU scores often reported on PHOENIX14T may be partially explained by the presence of repeated or highly similar sentences across training and test splits. This overlap can lead to memorization rather than true learning, especially in models trained on small datasets. In contrast, CSL-Daily exhibits less redundancy and appears to support more robust generalization. However, even in this case, caution is warranted when interpreting evaluation metrics.

Based on our analysis, we offer the following recommendations for future SLT research:

- Dataset selection. We encourage the community to move beyond PHOENIX14T and CSL-Daily and adopt larger, more diverse datasets such as How2Sign, CSL-News, and FLEURS-ASL. These resources offer broader linguistic coverage and greater potential for training scalable, multilingual SLT systems. These larger datasets can also be exploited for pre-training SLT systems.
- Gloss-free modeling. Given the prohibitive cost of gloss annotation at scale, we advocate for increased focus on gloss-free approaches. The large-scale datasets mentioned earlier do not include gloss annotations, and future datasets are unlikely to either. Despite the challenges posed by representation density (Ye et al., 2024), recent work has shown promising advances in this direction, even outperforming gloss-based methods in some cases (Li et al., 2025). These developments should encourage the community to pursue gloss-free modeling more actively.
- Leveraging LLMs. The integration of large language models (LLMs) into SLT pipelines is a promising direction. While some studies have reported limitations—such as LLMs dominating training or failing to capture visual features—others have demonstrated improvements using models like mT5, LLaVA, and ByT5. A complementary approach is to use LLMs as a data augmentation tool: Dal Bianco et al. (2025) show that generating target-text paraphrases with GPT-4 yields a modest but consistent BLEU improvement on PHOENIX14T, attributing the gain to reduced overfitting to rigid surface patterns—consistent with the memorization effects we document in Section 3. Further research is needed to identify best practices for combining visual and linguistic representations.
- New direction for novel datasets. Carefully generated datasets should aim at being more diverse, with the least amount of repetitions or patterns that may be discerning (for example, the same introduction). However, it is also true that this leads to small datasets that are designed with precision, which may not be enough to train large systems. However, we think the use of large datasets crawled from the web can be used or pre-training while the small and carefully designed datasets can be used for finetuning. That is, quality should be improved for small datasets in order to make them useful. Another useful point would be preparing different splits for ML tasks, for example signer-independent splits, to evaluate the overfitting to specific people.

## Data Availability

Publicly available datasets were analyzed in this study. This data can be found here: https://github.com/olatz87/SLT-Datasets/blob/main/README.md.

## Footnotes

1 Glosses are written labels for signs. They are used to produce simplified transcriptions of sign languages.

2 For example, *guten abend liebe zuschauer* “good evening dear viewers" appears 63 times in the training set and six times in the test set.

3
https://rapidfuzz.github.io/RapidFuzz/Usage/fuzz.html#ratio

4 The BLEU score we report for the TwoStream-SLT model on PHOENIX14T (27.76) is slightly lower than the value originally reported by the authors (28.95). This discrepancy arises from a preprocessing difference: the original evaluation includes a trailing whitespace and full stop at the end of each reference sentence, whereas we remove these elements from both reference and generated outputs to ensure consistent and fair comparison across models.
